# Radial Variability of Selected Physical and Mechanical Parameters of Juvenile Paulownia Wood from Extensive Cultivation in Central Europe—Case Study

**DOI:** 10.3390/ma16072615

**Published:** 2023-03-25

**Authors:** Karol Tomczak, Przemysław Mania, Marcin Jakubowski, Arkadiusz Tomczak

**Affiliations:** 1 Łukasiewicz Research Network, Poznań Institute of Technology, Center of Wood Technology, Winiarska 1, 60-654 Poznań, Poland; 2 Department of Forest Utilization, Faculty of Forestry and Wood Technology, Poznań University of Life Sciences, Wojska Polskiego 71 A, 60-625 Poznań, Poland; 3 Department of Wood Science and Thermal Techniques, Faculty of Forestry and Wood Technology, Poznań University of Life Sciences, Wojska Polskiego 38/42, 60-627 Poznań, Poland

**Keywords:** biomass production, fast-growing trees, mechanical and physical properties, plantation, Shang Tong Hybrid F1

## Abstract

The research on Paulownia cultivation and wood properties is up to date in many countries. However, there are no data on the properties of this wood defined on a microscale, on microtome samples. The main aim of this study was to find the best valorization path for the wood of Paulownia Shang Tong Hybrid F1 from an extensively cultivated plantation established in April 2017 in Poland by determining the tensile strength, the wood density, the strength-to-density ratio, and the modulus of elasticity on a cross-section of the trunk. The wood was collected from extensive plantation, where production is based on the natural resources of the habitat and ambient weather conditions, which is the opposite to the intensive cultivation model, which is the recommended model of Paulownia cultivation. The results of this study show that the mean density of the analyzed samples was approximately 210 kg/m^3^ when the mean value of the modulus of elasticity (MOE) was approximately 2400 MPa. The mean result for the tensile strength ratio to density was 11.25 km. In the case of anatomical structure, the increasing trend with age was noticed both in fiber and vessel characteristics. The study results provide unique data worldwide about Paulownia wood’s properties based on a cross-section of the trunk, from plantations cultivated in conditions which are not recommended by seedlings producers. The obtained data indicate that the Paulownia wood (examined) from the cultivation in this study has a technical quality similar to that of model-intensive agricultural plantations.

## 1. Introduction

Wood as a material has been used by humans for millennia. It is a biological material with an anisotropic structure and variable properties. Systematic research conducted for various years has led to the development of a plethora of modern materials derived from wood [1,2,3,4], as well as other lignocellulosic materials [5,6] and non-wood composites [7,8]. However, research on raw wood as a biological structure continues. Wood is a renewable material, and industry is increasingly interested in rapid production cycles. In this context, high expectations are placed on fast-growing tree species, such as Paulownia [9,10]. Paulownia is a genus of plants of the family *Paulowniaceae*, originating from China [11]. In the past twenty years, the cultivation of plants in this genus has gained significant interest in many European countries. *Paulownia* wood has properties similar to willow and poplar, but it stands out due to its exceptional growth rate under ideal conditions [12,13]. The initial introduction of *Paulownia* species can be traced back to China, where species such as *P. tomentosa* and *P. fortunei* were grown [9,14]. However, for various reasons, these species were unsuccessful. Of particular concern was the invasive nature of *P. tomentosa*, which had previously caused issues in the United States [15]. As a result, Paulownia hybrids were developed and eventually became the most widespread and successful forms of the species for commercial production. The most known hybrids are ‘Clon in vitro 112′ [9,16], ‘Cotevisa 2′, ‘Sundsu 11′ [17], and ‘Shan Tong’ [18,19]. In Poland, the most commonly described hybrids are Clon in vitro 112 and Shan Tong [10]. It should be noted that Clon 112 is a registered hybrid reproduced in vitro from a single genotype [20,21], while for Shan Tong, no specific genotype is registered. Paulownia hybrids are typically cultivated for short cycles of 6–8 years [16]. Prolonged experiments in southern Europe have demonstrated the cost-effectiveness of cultivation [17,22]. In contrast, cultivation in central and eastern Europe is at a very early stage, and there is no long-term confirmation of its effectiveness and profitability [20,23]. Nonetheless, *Paulownia* wood is gaining popularity, and several studies on its properties have been undertaken [24,25,26].

Because of the diversity of growth and development conditions of the species and its hybrids, Paulownia exhibits high variability in its wood properties [24,27,28,29]. For instance, wood density can range from 220 to 350 kgm^−3^, although it is usually close to 270 kgm^−3^ [24,27,28]. Different researchers have obtained widely varying results for the same species: reported values of *P. tomentosa* wood density range from 268 kgm^−3^ [30] to 317 kgm^−3^ [27], while values of its static flexural strength range from 3492 MPa [24,28] to 4281 MPa [27]. The Clon 112 hybrid, however, exhibits smaller variation [31,32].

Research into the properties of wood is crucial for its applications; however, in the case of *Paulownia*, it is mainly important for planning biomass production. The outcomes of biomass production are mainly studied in countries with extensive experience in cultivation, particularly Spain [17,33], Portugal [34], Romania [35], Iran [36], Serbia [13], and other countries mostly in Southern Europe.

Every type of wood exhibits specific variability along a radial cross-section, which impacts subsequent uses of the wood. Coniferous species usually exhibit an increase in density and mechanical properties from the core to the perimeter [37,38,39]. Conversely, in ring-porous species, the opposite trend is found [40,41,42]. Diffuse-porous species, on the other hand, display significant variation in their properties, with no clear upward or downward trend [43,44]. *Paulownia* species are ring-porous or semi-ring-porous [11,45], with a regular arrangement of vessels in earlywood and fibers in latewood. Such wood can be expected to exhibit a trend dependent on the distance from the core. However, it is difficult to investigate such patterns in young plantation-grown trees in view of the very wide annual increments. Results for radial variability obtained for standard-sized samples (with cross-sections 2 cm × 2 cm) will always be subject to a certain error, resulting from the non-uniform proportions of earlywood and latewood in samples with very wide annual rings. To overcome this challenge, it is necessary to analyze a large number of rings, which can only be accomplished with very old trees or by using non-standard research methods involving the testing of microsections, which was used in this study. Knowledge of radial variation can help predict biomass yields for specific wood dimensions.

The cultivation of trees of the family *Paulowniaceae* in Poland and worldwide typically follows an intensive agricultural model, involving regular fertilization and irrigation to maximize biomass yields. An alternative model that adheres to sustainable agriculture principles is extensive production based on natural resources of the habitat and ambient weather conditions. Nevertheless, its productivity and impact on wood properties have not yet been studied. While Paulownia wood has been studied in many world-renowned research centers, there is a lack of data on the properties of this wood defined on a microscale, on microtome samples. Research on the radial variability of wood parameters can provide insights into how the strength of wood changes within annual growth rings. The present work aimed to determine the radial variability of the modulus of elasticity, tensile strength, and density of juvenile wood of *Paulownia* Shang Tong Hybrid F1 from an extensively cultivated plantation in Poland to identify the best valorization path. The properties investigated and analyzed included the tensile strength (TS) distribution along microtome fibers of samples from all the annual growth rings, the variability of the wood density, the strength-to-density ratio, and the linear modulus of elasticity in all annual growth rings.

## 2. Materials and Methods

### 2.1. Study Design

**Figure 1:**
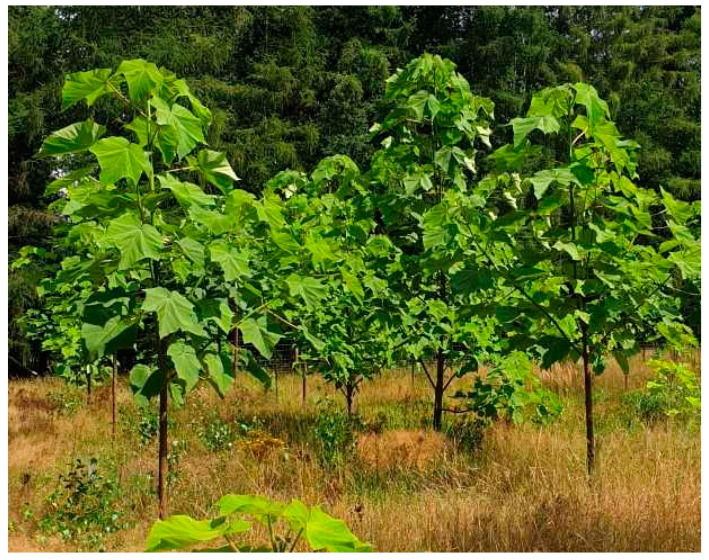
Overview of the plantation.

The study was conducted on raw wood material collected in 2019 from a *Paulownia* plantation established in April 2017 at the Forest Experimental Station in Murowana Goślina, near Poznań, Poland. The plantation was established on forest soil of the podzolic type with the planting of one-year-old seedlings. The plantation is located in a zone influenced by a temperate climate of a transitional nature. The average annual temperature of the analyzed area is 8.2 °C, while the average annual precipitation ranges between 500 and 530 mm and is lower than the national average. The length of the growing season is 225 days. The average height of the trees was 2 meters, the average diameter at breast height was 5 cm, and the diameter of stems at 5 cm height was approximately 7 cm (Figure 1). The research material was cut wooden planks from two different Paulownia trees, obtained from breast height and ground level.

### 2.2. Characteristics of Raw Material and Anatomical Structure

**Figure 2:**
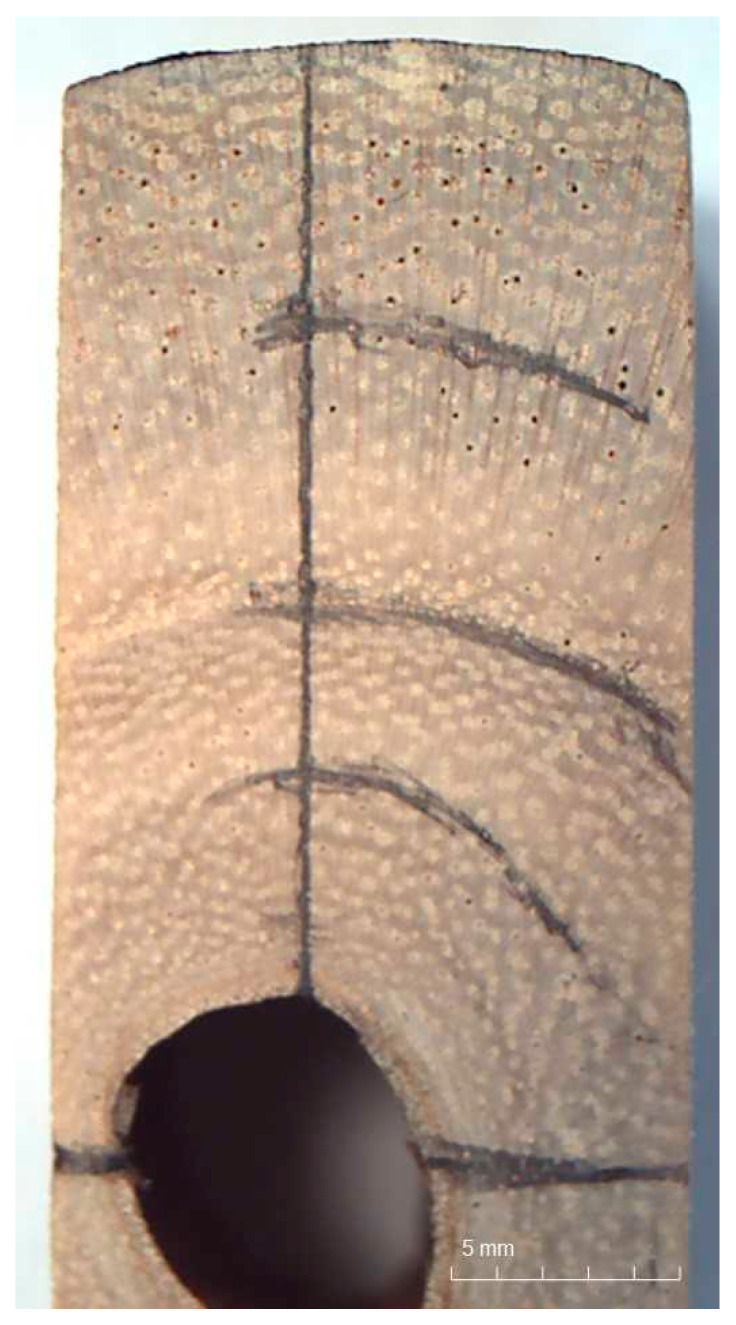
Cross-section of a young Paulownia tree trunk with a marked macroscopic structure.

After the trees were collected from the plantation, the width of the annual rings was marked and then measured by using a stereoscopic microscope (Figure 2). The average width of the annual ring in first tree was 5.93 mm, while in the second tree, it was 6.8 mm (Table 1).

The basic physical parameters of this wood were also determined. The maximum swelling of wood was determined on samples containing all of the analyzed annual growth rings. Dry samples obtained from the boards were placed in water until the sample reached its maximum volume. Swelling in the longitudinal direction was 0.28%, in the radial direction, it was 1.99%, and in the tangential direction, it was 5.67%. Twin samples were placed in a desiccator above water. Relative air humidity oscillated around 100%. The samples were conditioned until the weight stabilized. Then, the value of the fiber saturation point (FSP) for the tested wood was calculated. The FSP was around 29.8%.

After macroscopic measurements, the material was prepared to determine the anatomical features of the wood. To prepare samples for fibers and vessel elements length measurements, the material was collected separately from the investigated annual rings and was then subjected to tissue maceration. For this purpose, a 1:1 acetic acid: hydrogen peroxide mixture was used. Maceration was run in an incubator at 60 °C for 24 h. Each annual ring’s lengths of 30 fibers and vessel elements were measured, and the mean lengths were calculated.

**Figure 3:**
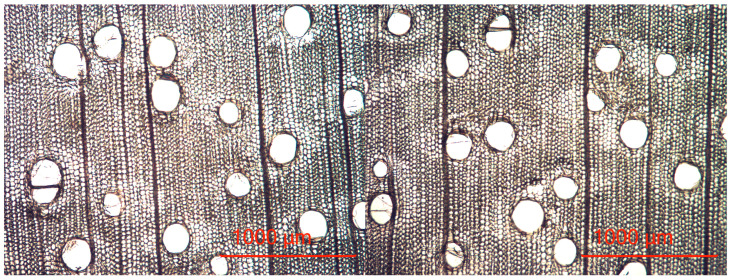
Cross-section of the analyzed wood tissue.

Paulownia belongs to a species of deciduous trees of a ring-porous or semi-ring-porous structure, depending on the species and the place of occurrence and growth conditions. The analyzed case’s first two annual growth rings can be classified as semi-ring-porous wood. Vessels of a fairly similar diameter occur across the width of the entire annual growth ring (Figure 3).

### 2.3. Estimation of Wood Properties

**Figure 4:**
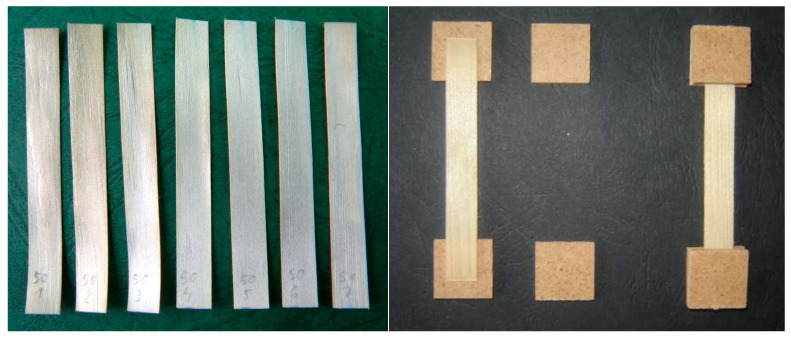
Sliced microtome samples and samples with reinforced grips.

The thickness of the planks in the tangential direction was 10 mm. Due to the need to plasticize the wood for later cutting, the boards were soaked in distilled water at room temperature for two months. The initial cutting tests confirmed that this wetting period was sufficient to enable the cutting of the desired thickness of microtome samples from the board without additional heating. Next, appropriate microtome samples for use in the tests were cut tangentially. The pieces had a radial thickness of approx. 200 µm allowing for several samples from single annual increments to be obtained. Slicing was carried out using a Leica Histoslide 2000 microtome sledge. Sliced pieces were placed sequentially on filter paper, annotated for later identification (Figure 4), and then conditioned in the laboratory until an equilibrium moisture was obtained. The samples were sliced from the core to the bark and taken from the entire wood ray. From the first tree, 90 samples from the ground section (IG) and 47 from breast height (IC) were obtained, and from the second tree, the numbers of samples were 91 and 40, respectively (IIIG and IIIC). The moisture content of the samples obtained was about 8%.

The wood density of each sample was determined by the stereometric method. The thickness of the samples was measured with a micrometric screw with an accuracy of 0.001 mm, in three places: at mid-length and a distance of 20 mm from the ends of the samples. The width of the samples was measured at mid-length with an accuracy of 0.1 mm using a Brinell magnifier and the length with an accuracy of 1 mm with a linear ruler. The weight of each test sample was determined using a laboratory balance with an accuracy of 0.001 g.

Before the tensile test, the gripped parts of specimens were glued with hardboard sections with dimensions of 20 × 20 mm and a thickness of 3 mm (Figure 4), using Pattex Wood Express glue. This was carried out to protect the samples against damage from the holders of the testing machine.

Tensile testing of the samples was carried out on a Zwick Z050TH testing machine using a Zwick 066550.02 extensometer with the base of a 30 mm extensometer. The test was performed at a speed selected so that the entire test lasted 60 ± 30 s and a preload of 10 N was applied. The samples were stretched along the fibers. The testing machine’s software indicated the maximum stress, which is the tensile strength of the wood along the fibers and the MOE.

Based on the results for the stereometric density and modulus of elasticity, the strength-to-density coefficient (SQC) was calculated according to the following Equation (1):

(1) SQC=MOE/D

where SQC is the strength-to-density coefficient, MOE is the modulus of elasticity, and D is the stereometric density.

### 2.4. Statistical Analyses

In the first step, to verify the distribution of the data, the Shapiro–Wilk test was performed. The data met the requirements of normality. To compare the data between samples from the ground section, from breast height, and between annual rings, a Student’s *t*-test was performed. Then, the Pearson correlation between the examined properties was determined. The RStudio program and R package 4.2.1 (R Core Team 2022, Vienna, Austria) were used for the calculations.

## 3. Results

### 3.1. Morphological Parameters

The mean length of the fibers was app. 803 μm; short fibers were noticed in samples collected from the 1st annual rings, while the longest fibers were noticed in the samples from the 4th rings. The trend of increasing the fiber length with the age of the tree was observed. The mean vessel length was app. 230 μm, while the mean vessel diameter was app. 157 μm. An increase in the size of the vessels with age was noted (Table 2).

### 3.2. Mean Statistics of Examined Properties

The samples collected at breast height were denser than the samples from the ground section. The greatest density was observed in the case of the samples from sections IC and IIIC (approx. 229 kg/m^3^). The Student’s t-test showed statistically significant differences between the samples from sections IG and IIIG. The mean density for all the collected samples was approximately 211 kg/m^3^ (Table 3). In the case of the tensile strength (TS), the greatest value (16.23 MPa) was observed for the samples collected from section IIIC, while the lowest value was observed for samples collected from section IIIG. In tree I, we observed a different variation in TS. The TS was greater in IG than in IC. The mean TS was approximately 13 MPa. Statistically significant differences were found only in the case of samples IG and IIIG (Table 4). The largest MOE (3034.5 MPa) was observed for IIIC, similar to the IC value (approx. 3025 MPa). The lowest value was recorded for IG. The mean MOE was approximately 2401 MPa. There were no statistically significant differences in the MOE between the examined samples (Table 5). In the case of the strength-to-density coefficient, greater values were obtained at breast height (IC and IIIC), and a lower SQC was observed in samples collected from sections IG and IIIG. The differences between the examined samples were not statistically significant (Table 6).

### 3.3. Distribution on the Cross-Section of the Trunk

**Figure 5:**
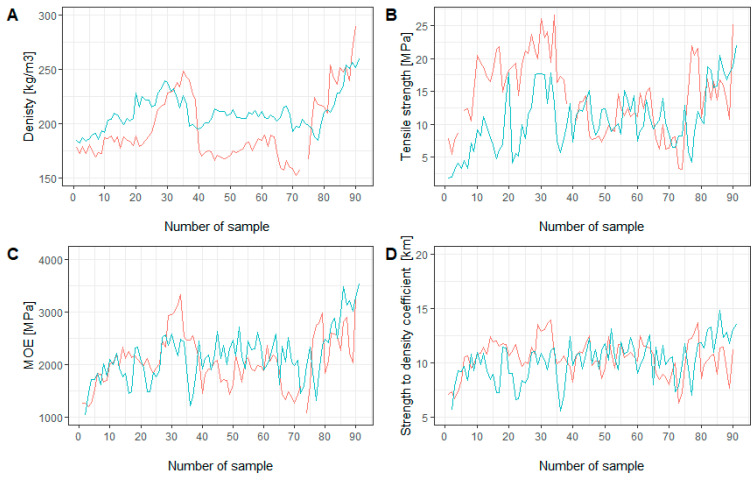
Cross-section comparison of examined properties from the ground section: (**A**) density [kg/m^3^]; (**B**) modulus of rupture [MPa]; (**C**) modulus of elasticity [MPa]; (**D**) strength-to-density coefficient [km]. Sample no. 0 is from the pith, and 90 is at the bark. IG (red line): the first annual ring between samples 1 and sample 34, the second annual ring between samples 35 and 64, and the third annual ring between samples 65 and 90. IIIG (blue line): the first annual ring between samples 1 and sample 29, the second annual ring between samples 30 and 67, and the third annual ring between samples 68 and 91.

**Figure 6:**
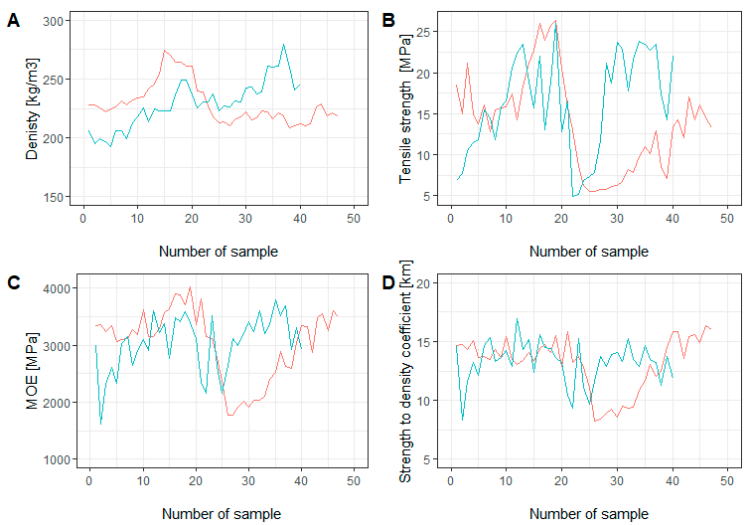
Cross-section comparison of examined properties collected at breast height: (**A**) density [kg/m^3^]; (**B**) tensile strength [MPa]; (**C**) modulus of elasticity [MPa]; (**D**) strength-to-density coefficient [km]. Sample no. 0 is from the pith, no. 90 is at the bark. IC (red line): the first annual ring between samples 1 and sample 15 and the second annual ring between samples 16 and 47. IIIC (blue line): the first annual ring between samples 1 and sample 19 and the second annual ring between samples 20 and 40.

The distribution of the examined properties over the trunk cross-section was very diverse. In the case of the samples taken from the ground section, the lowest values of D, TS, MOE, and SQC were observed close to the pith, and then dynamic changes were observed in the direction of the bark. In both cases, similar trends were observed (Figure 5A–D). The distribution of the studied properties at breast height differed from that in the ground section. Nevertheless, similar trends were noticed in the case of the TS, MOE, and SQC. The density distribution in samples IC and IIIC followed a similar course from the pith to the middle part of the cross-section, after which the value for IIIC increased, while that for IC remained at the same level (Figure 6A–D).

### 3.4. Pearson Correlation between Examined Properties

**Figure 7:**
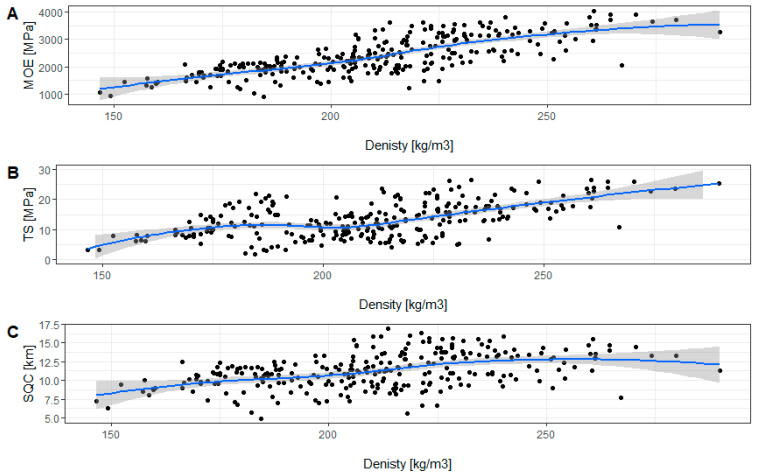
Correlation of density with the modulus of elasticity (**A**), tensile strength (**B**), and strength-to-density coefficient (**C**) for all collected samples.

We found a strong positive correlation between density and the MOE (0.75) and TS (0.54) for all samples examined in our study (Figure 7A–C). The results of the Pearson correlation for samples from the ground section show a strong positive correlation between density and the modulus of elasticity (0.71), between the MOE and tensile strength (0.74) and the SQC (0.86), and between the SQC and TS (0.68). The correlations between D and TS and between D and SGC are also positive but not as strong as in the other cases (Table 7). In the case of samples collected at breast height, strong positive correlations were found for every pair of properties. The highest correlation was found between the SQC and MOE (0.90) and between the TS and MOE (0.74), and the weakest correlation was found between the SQC and D. The correlation between the TS and D (0.66) was much higher at breast height than in the ground section (Table 8).

### 3.5. Mean Statistics for Examined Properties Divided by Annual Rings

The greatest density value was observed in the wood samples collected from annual ring II. An increase in the value of D was observed in a direction from pith to bark; however, the value decreased closest to the bark. The results of the Student’s *t*-test showed no statistical differences in density between the rings. The same phenomenon was observed for the MOE, although a statistically significant difference in the MOE was observed between rings II and III. Similar results as for the MOE were obtained in the case of the SQC, where a statistically significant difference was identified only between the second and third rings. In the case of TS, the highest value was recorded in ring I, and then a decrease was observed in the direction towards the bark. A statistically significant difference in TS was detected between rings I and III (Table 9).

## 4. Discussion

The results of this study show the density (D) of analyzed samples of the wood of *Paulownia* Shang Tong Hybrid F1 to be approximately 210 kg/m^3^. This is a typical value for *Paulownia*, enabling the wood to be categorized as very light [9,31]. Jakubowski [45] states, based on a review of the subject literature, that the density of *Paulownia* wood with a 12% moisture content ranges from 220 to 350 kg/m^3^ and is usually close to 270 kg/m^3^. The value obtained in this study is 28% lower than that. It is possible that, besides species characteristics, the result was influenced by the relatively young age of the model trees from which samples were taken. *Paulownia* hybrids display very rapid increments in thickness. The part of the cross-section next to the core will have typical features of juvenile wood [46]. In fast-growing species cultivated on plantations, the differences in structure and properties between juvenile and mature wood are well known [47,48,49,50].

Juvenile wood is a natural feature of the tree structure and occurs in all species of trees. It is usually found within up to 20 annual rings in the core’s vicinity [46]. This depends on the tree species; plantation species usually have a higher proportion of juvenile wood. Trees on plantations are usually harvested after 30 years, so approximately half of the wood produced comes from the young part of the trunk [51]. This is a problem because a high share of juvenile wood is associated with the reduced strength of the timber, problems with warping during the drying process, and lower yields in the production of cellulose mass [52]. The width of the juvenile wood in paulownia is not precisely defined. One criterion for considering wood as mature is the properties of the wood. In the study described, the upward trend in both density and MOE and TS is weak, although present. It is likely that the wood is still juvenile and the wood tissue is maturing. At this stage of the study, it is difficult to determine the maturity of the wood tissue. We expect this will be possible in the next few years. The juvenile nature of the wood is indicated by the low results of the parameters obtained including density, which ranged from 193 to 229 kg/m^3^. Similar results for juvenile wood of Shan Tong paulownia were reported by Sedlar et al. [18]. Here, a density of 220–233 kg/m^3^ was obtained for a 4-year-old plantation established in the Republic of Croatia. In addition, San et al. [53] reported a low-density value (214 km/m^3^) for 3-year-old paulownia wood grown in China. Fos et al. [54] noticed that in the case of Paulownia hybrid Cotevisa 2, the transition from juvenile to mature wood occurs from the 5th year of growth. The differences between the mean density of the first four annual rings and the fifth and sixth were 41 kg/m^3^. In comparison to Fos et al. [54], the width of annual rings obtained in this study was much narrower. In the case of trees collected in our study, the 1st annual ring had app. 5.2 mm, while in Fos et al.’s [54] study, it was over 40 mm, then the average ring width was decreased and stabilized after the 5th year. When it comes to the wood structure of wood from extensive cultivation, there is no such trend, the widest annual rings were noticed in the 3rd year—app. 9.3 mm, and the narrowest were in 1st year 4.61 mm. These differences in tree growth can be caused by different growth conditions, such as the type of cultivation, weather conditions, and the length of the growing season. Hussain et al. [55] showed that the average width of the annual ring also depends on the Paulownia species. The authors found the widest annual rings in *P. catalpifolia* and *P. fortunei*—over 16 mm—while *P.tomentosa* was characterized by the width of rings around 6.5 mm. The measured wood was obtained from clone ‘Shan Tong’ Hybrid F1; this variety is a hybrid of P.tomentosa and P. fortunei. The average 6.37 mm was obtained. The fiber length obtained in our study ranged between 749 and 871 μm, with the vessel length ranging between 212 μm up to 246 μm and its diameter ranging between 147 and 171 μm. Ashori and Nourbakhsh [56] noticed a fiber length from 873 to 1150 μm for 6-years-old Paulownia; however, the authors did not divide the results by rings. When Yue et al. [57] studied the anatomical characteristics of tension, lateral, and opposite branch wood, they noticed a fiber length between 881 and 749 μm and a vessel diameter from 239 to 195 μm. In our study, the increasing trend with age was noticed both in fiber and vessel characteristics.

The density of wood is correlated with its mechanical properties. However, the strength of this correlation may depend on the specific species or hybrid. Lachowicz and Giedrowicz [25] state that, in comparison with other species, the wood of *Paulownia* COTE-2 has greater mechanical strength despite its lower density. This is a highly favorable configuration from the point of view of potential uses of the wood. We found a strong positive correlation with density for both the TS and MOE. However, by analyzing individual data sets, depending, for example, on the position of samples along the trunk, the correlation was weaker. The correlation between D and MOE is generally stronger than between D and TS.

Mechanical property tests are mainly performed on mature wood, which is characteristic of older trees. This is mainly because of the possibility of obtaining a suitable sample size. Higher results can be seen mainly in mature wood. Depending on the species, the reported values of the MOE range from approximately 2600 to 5900 MPa [27,28,31,53,58]. We obtained lower values than this. The mean MOE was 2400 MPa, and for IG, the mean MOE was approximately 2050 MPa. The values of the TS were similarly lower: the mean for all samples was 13.24 MPa, while Koman et al. [58] obtained a value for P. tomentosa of 33.23 MPa.

The average result for the tensile strength-to-density ratio was 11.25 km. This ratio is a good indicator for comparing wood with other materials. For example, balsa wood has excellent ratios of rigidity to mass and strength to mass [59]. Generally, wood has a higher strength-to-density coefficient than other materials.

Various studies of the wood of *Paulownia* are based on the material obtained from very young trees to find the best valorization path [60]. Perhaps for this reason there is little information in the literature about the radial variation in the properties of the wood. Sánchez-Machado et al. [61] showed, in a study of material obtained from a five-year-old plantation of *P. tomentosa*, that the density of the wood increased from core to bark. The difference in density at the core and at the bark was approximately 200 kg/m^3^. Such a distribution of values along the radius is typical of coniferous species [62,63]. Our analysis concerned material obtained from a three-year-old plantation, and the adopted methods made it possible to obtain numerous samples from each annual ring. A comparison of successive annual rings showed no increase in D with cambial age. The mean values of D within particular rings were very similar, close to the mean value for all samples. Similar distributions of values were obtained for the TS and MOE. However, by analyzing the distribution of values in particular rings, it was observed that both density and strength increased. This applies in particular to the first annual ring. The increase is not abrupt; thus, it is not possible within an annual ring to distinguish earlywood and latewood zones, which is a typical feature of ring-porous species. According to Zhu et al. [11] and Qi et al. [64], *Paulownia* species are ring-porous or semi-ring-porous in view of the regular distribution of vessels in the earlywood and fibers in the latewood. In our analysis, the density of the wood differed significantly between the start and the end of the vegetative period. In the case of tensile strength, the spread of values was greater. A similar phenomenon was observed for the MOE, whereas the strength-to-density coefficient was more uniform.

Considering Paulownia’s rapid growth in thickness, the large variation in properties within an annual ring may pose problems in the practical applications of wood. Wood is, by nature, a heterogeneous and anisotropic material. Heterogeneity may be defined as differences in the structure and properties of the wood at both sub-microscopic and macrostructural levels, for example, the cell wall structure and the structure and properties of earlywood in an annual ring. Fast-growing species require detailed analysis in this respect, in view of the increasing interest in their cultivation.

## 5. Conclusions

This work aimed to determine the properties of wood on the cross-section of the trunk from very young trees of the Paulownia Shang Tong Hybrid F1 cultivated on a plantation in Poland without intensive treatments in order to identify the best valorization path. Although research about Paulownia cultivation and wood properties is up to date in many countries, in the literature on the subject, there are no data on the properties of this wood defined on a microscale, on microtome samples. Moreover, there are no available data about the extensive cultivation of Paulownia trees in any scientific issue. In general, experiments on Paulownia cultivation are usually focused on their production and based on an intensive agricultural model.

The results of this study show that the mean density of the analyzed samples was approximately 210 kg/m^3^, while the mean MOE was approximately 2400 MPa. The mean result for the tensile strength ratio to density was 11.25 km. In the case of anatomical structure, the increasing trend with age was noticed both in the fiber and vessel characteristics. This may indicate the maturation of juvenile wood. Only in the case of the annual ring width, there were no observations indicating a stabilization of growth.

This study’s results provide unique data worldwide about Paulownia young wood’s properties from plantations cultivated in conditions that are not recommended by seedlings producers and show that it is possible to obtain good quality Paulownia wood from extensive cultivation in this region.

## Figures and Tables

**Table 1:** Width of the annual rings of the examined Paulownia tree trunks.

Annual Ring	I Tree (IG)	II Tree (IIIG)
I	5.78	4.61
II	4.94	4.76
III	7.22	9.33
IV	5.76	8.51
Mean	5.93	6.80

**Table 2:** Morphological features in the analyzed growth rings.

Anatomical Feature	Growth Ring Number				Mean Value
	1	2	3	4	
Fiber length [μm]	748.7 ± 109.2	779.5 ± 112.6	814.0 ± 119.0	871.5 ± 126.7	803.43
Vessel length [μm]	212.4 ± 48.9	220.8 ± 50.4	231.5 ± 53.7	246.4 ± 56.8	230.03
Vessel diameter [μm]	147.2 ± 36.2	151.6 ± 37.3	160.5 ± 39.5	170.8 ± 42.0	157.52

**Table 3:** Basic statistical parameters for the density [kg/m^3^] and results of the Student’s *t*-test for samples collected from the ground section (G) and breast height (C) from both trees.

	Mean	N	Std. Dev	Var	Min	Max	Median	Student’s *t*-Test
IG	193.98	90	30.44	926.55	146.59	289.74	183.43	0.00
IIIG	210.20	91	16.76	280.98	182.29	260.08	207.03	
IC	229.11	47	17.35	301.06	208.66	274.20	224.54	Ns*
IIIC	229.24	40	20.42	417.02	192.20	279.76	228.71	
Mean	210.91	268	26.87	721.83	146.59	289.74	211.78	

Ns*—not statistically significant.

**Table 4:** Basic statistical parameters for the tensile strength [MPa] and results of the Student’s *t*-test for samples collected from the ground section (G) and breast height (C) from both trees.

	Mean	N	Std. Dev	Var	Min	Max	Median	Student’s *t*-Test
IG	14.25	87	5.51	30.40	3.11	26.60	13.90	0.00
IIIG	10.65	91	4.60	21.15	1.83	22.00	10.10	
IC	13.87	47	5.86	34.40	5.48	26.40	14.20	Ns
IIIC	16.23	40	6.09	37.11	4.88	26.00	16.50	
Mean	13.24	265	5.72	32.67	1.83	26.60	12.90	

**Table 5:** Basic statistical parameters for the modulus of elasticity [MPa] and results of the Student’s *t*-test for samples collected from the ground section (G) and breast height (C) from both trees.

	Mean	N	Std. Dev	Var	Min	Max	Median	Student’s *t*-Test
IG	2049.53	90	506.56	256,607.02	938.00	3330.00	2025.00	Ns
IIIG	2147.13	91	504.31	254,328.36	899.00	3530.00	2110.00	
IC	3025.74	47	619.01	383,168.46	1770.00	4030.00	3160.00	Ns
IIIC	3034.50	40	489.88	239,984.36	1620.00	3800.00	3115.00	
Mean	2400.88	268	681.74	464,765.68	899.00	4030.00	2305.00	

**Table 6:** Basic statistical parameters for the strength-to-density coefficient [km] and results of the Student’s *t*-test for samples collected from the ground section (G) and breast height (C) from both trees.

	Mean	N	Std. Dev	Var	Min	Max	Median	Student’s *t*-Test
IG	10.49	90	1.63	2.65	6.29	13.99	10.67	Ns
IC	13.15	47	2.29	5.24	8.27	16.32	13.67	
IIIG	10.16	91	1.93	3.72	4.87	14.81	10.32	Ns
IIIC	13.23	40	1.80	3.22	8.31	16.90	13.46	
Mean	11.25	268	2.31	5.34	4.87	16.90	11.07	

**Table 7:** Pearson correlation of examined properties from the ground section: density, modulus of elasticity, tensile strength, and strength-to-density coefficient.

	D	MOE	TS	SQC
D	x	0.71	0.46	0.26
MOE	0.71	x	0.74	0.86
TS	0.46	0.74	x	0.68
SQC	0.26	0.86	0.68	x

**Table 8:** Pearson correlation of examined properties at breast height: density, modulus of elasticity, tensile strength, and strength-to-density coefficient.

	D	MOE	TS	SQC
D	x	0.60	0.66	0.19
MOE	0.60	x	0.74	0.90
TS	0.66	0.74	x	0.53
SQC	0.19	0.90	0.53	x

**Table 9:** Basic statistical parameters of the density [kg/m^3^], tensile strength, modulus of elasticity [MPa], and strength-to-density coefficient [km] of samples divided by annual rings, from the pith (I) to the bark (III).

Ring	Mean	N	Std. Dev	Var	Min	Max	Median
Density [kg/m^3^]							
I	207.98	98	23.04	531.06	2.33	187.04	206.04
II	213.55	120	25.45	647.59	2.32	199.88	212.77
III	210.31	50	35.70	1274.61	5.05	188.25	210.53
Mean	210.91	268	26.87	721.83	1.64	189.38	211.78
Tensile strength [MPa]							
I	14.25	97	6.33	40.10	0.64	8.60	15.00
II	12.65	118	5.17	26.78	0.48	8.53	12.05
III	12.70	50	5.51	30.32	0.78	8.23	12.40
Mean	13.24	265	5.72	32.67	0.35	8.43	12.90
Modulus of elasticity [MPa]							
I	2378.66	98	711.87	506,753.46	71.91	1840.00	2235.00
II	2481.25	120	651.89	424,960.61	59.51	2000.00	2340.00
III	2251.56	50	676.56	457,729.03	95.68	1620.00	2295.00
Mean	2400.88	268	681.74	464,765.68	41.64	1910.00	2305.00
Strength-to-density coefficient [km]							
I	11.30	98	2.60	6.76	4.87	16.90	11.22
II	11.52	120	2.13	4.55	5.55	16.32	11.17
III	10.54	50	1.99	3.94	6.29	14.81	10.60
Mean	11.25	268	2.31	5.34	4.87	16.90	11.07

## Data Availability

The datasets generated and/or analyzed during the current study are available from the corresponding author ipon reasonable request.
